# Characterization and postprandial regulation of the nutrient-sensing machinery in the oral floor mucosa of 10-week-old mule ducks

**DOI:** 10.1016/j.psj.2026.107806

**Published:** 2026-09-21

**Authors:** Marie Lasserre, Yan Fericelli, Cécile Heraud, Laura-Lou Zwick, Karel Sasso, Sandra Biasutti, Anne Surget, Joelle Dupont, Marianne Houssier, Stéphane Panserat, Jérôme Roy, Karine Gontier

**Affiliations:** a Université de Pau et des Pays de l’Adour, INRAE, NUMEA, Mont-de-Marsan, France; b Université de Pau et des Pays de l'Adour, INRAE, NUMEA, Saint-Pée-sur-Nivelle, France; c Université de Tours, INRAE, CNRS, UMR 7247 PRC, Nouzilly, France

**Keywords:** Avian chemosensation, Postprandial response, Oral floor mucosa, Neurotransmitters turnover, Hypothalamus

## Abstract

In birds, oral nutrient detection contributes to regulating ingestive behavior, yet the molecular repertoire and postprandial dynamics of gustatory pathways remain poorly defined in ten-week-old ducks. This study evaluated the postprandial responses of nutrient-sensing receptors, calcium-signaling components, and monoaminergic pathways in the oral floor mucosa and central neural tissue of ten-week-old mule ducks. Fed and fasted ducks were sampled at 0 min, 20 min, 2 h, 6 h, and 12 h following meal distribution. Transcriptional profiling revealed a broad chemosensory repertoire spanning sweet, umami, bitter, sour, and fatty acid modalities in the oral floor mucosa, supporting its role as a key sensory interface. However, most gustatory and calcium-signaling genes exhibited remarkable transcriptional stability postprandially at this developmental stage. Notably, only the lipid sensor *CD36* (Cluster of differentiation 36) and the bitter receptor *T2R116* (Taste receptor type 2 member 116) displayed a significant time × feeding interaction, indicating targeted peripheral responsiveness for fat and bitter perception. Conversely, postprandial changes were marked in monoaminergic circuits. Serotonin metabolism in the oral floor mucosa varied by nutritional state during the early postprandial phase, while elevated central serotonin concentrations and enhanced dopaminergic turnover in fed ducks evidenced acute activation of central satiety and reward circuits. Concurrently, hypothalamic gene expression revealed a synchronized time-dependent downregulation of *POMC* (Proopiomelanocortin) and *MC4R* (Melanocortin 4 receptor) up to 6 h post-feeding. In parallel, plasma chemerin levels increased postprandially, highlighting a coordinated peripheral metabolic response. Overall, these results indicate that short-term postprandial regulation in ten-week-old ducks relies predominantly on rapid neurochemical adjustments rather than transcriptional remodeling of peripheral gustatory receptors.

## Introduction

The regulation of feed intake undergoes continuous adaptations during growth to align with changing physiological demands (Richards and Proszkowiec-Weglarz, 2007). In waterfowl production, these adaptations coincide with major dietary shifts, notably transitioning from a high-protein starter diet to a lower-protein, higher-energy grower feed after four weeks of age (Fouad et al., 2018). Such transitions demand regulatory flexibility, particularly as birds approach the pre-force-feeding stage in traditional foie gras production (Bonnefont et al., 2019).

In ten-week-old mule ducks, this developmental stage represents a critical physiological window prior to traditional force-feeding for foie gras production. At this maturity level, feed intake is under tight homeostatic control, enabling ducks to adjust consumption according to metabolic needs. Unlike geese, ducks exhibit strong resistance to spontaneous hyperphagia due to stringent central and peripheral intake controls (Bley and Bessei, 2008; Guy et al., 2013). This homeostatic control involves tight coordination between peripheral chemosensory signals and central nervous system. In addition, feeding behavior in ducks displays individual variability and dynamic meal patterns, reflecting species-specific regulatory strategies (Richards and Proszkowiec-Weglarz, 2007). Characterizing these mechanisms is therefore essential to identify the sensory and neurochemical pathways that govern feeding behavior. Ultimately, this knowledge could contribute to reducing the reliance on force-feeding by promoting voluntary intake levels that meet production standards while supporting animal welfare.

Within this framework, peripheral gustatory cues provide immediate feedback on dietary nutrient composition, serving as the first line of ingestive control (Roura et al., 2013; Niknafs et al., 2023). In birds, taste buds are predominantly distributed across the palate and oral mucosa rather than the keratinized tongue (Kudo et al., 2008). Previous work in younger ducks (4 weeks of age) highlighted the oral floor mucosa as a highly responsive chemosensory interface, displaying pronounced postprandial modulation of gustatory receptors (Lasserre et al., 2026).

Peripheral sensory signals are integrated at the central level to influence feeding behavior. Peripheral chemosensory signals originating from the oral floor mucosa project to central feeding circuits, most notably the hypothalamus, which act as the primary integrator of feeding behavior and energy homeostasis (Schwartz et al., 2000; Morton et al., 2006). This integration involves central mediators such as serotonin and dopamine, which contribute to satiety- and reward-related pathways, alongside neuropeptidergic networks comprising orexigenic Agouti-related peptide (**AgRP**) and neuropeptide Y (**NPY**) and anorexigenic proopiomelanocortin (**POMC**) and cocaine- and amphetamine-regulated transcript (**CART**) signals that regulate energy balance and feeding responses (Berthoud, 2004; Arora and Anubhuti, 2006; Donovan and Tecott, 2013). In four-week-old mule ducks, these neuropeptides were expressed but not transcriptionally regulated in response to a meal, as previously shown in our earlier study. By contrast, at the protein level, postprandial activation of serotonergic and dopaminergic pathways was detected, suggesting that acute central responses to feeding at this stage are mediated primarily through neurotransmitter signaling rather than changes in neuropeptide gene expression (Lasserre et al., 2026).

Developing viable alternatives to traditional force-feeding via voluntary hyperphagia requires addressing a critical knowledge gap: how the oral floor mucosa and central circuits coordinate meal responses at later developmental stages, i.e., around 10 weeks of age. At this stage, how the buccal floor mucosa responds to meal ingestion remains poorly understood, highlighting a critical knowledge gap that limits the development of strategies aimed at enhancing spontaneous intake under farming conditions.

Because feeding regulatory mechanisms may evolve with physiological maturation, understanding how these processes operate at later developmental stages is essential. Therefore, this study aimed to characterize the molecular and sensory mechanisms underlying feed intake regulation in ten-week-old mule ducks. Building on our previous work in four-week-old ducks (Lasserre et al., 2026), we provide here a more comprehensive molecular characterization by expanding the panel of target genes associated with the five taste modalities and intracellular calcium signaling. We hypothesized that, at ten weeks of age, ducks would display a more mature integrative response, characterized by coordinated postprandial regulation across both peripheral sensory receptors and central neuropeptidergic pathways that were previously unresponsive in younger birds.

Specifically, we analyzed the postprandial effects of a standardized meal on this expanded panel of peripheral markers in the buccal floor mucosa, together with central neurochemical and neuropeptidergic responses, to better decipher feed intake regulation at this pivotal pre-force-feeding stage.

## Materials and methods

### Animals and experimental procedures

All experimental protocols strictly complied with French national regulations on research animal care and were approved by the local Animal Ethics Committee at the INRAE Waterfowl Breeding Experimental station (Artiguères, France; accreditation B40-037-1). Male mule ducks (*n* = 180; MMG AS × PKL; MULOR, Carcarès-Sainte-Croix, France) were reared under standardized conditions. Birds received a commercial starter diet until 4 weeks of age (PAG1301; 17.5% crude protein, 2.85% crude fat, 43.52% starch, 2,800 kcal/kg metabolizable energy), and were subsequently switched to a commercial grower diet until 10 weeks of age (PAG1320; 15% crude protein, 2.25% crude fat, 48.52% starch, 2,849 kcal/kg metabolizable energy) (Table 1).

**Table 1:** Composition of the experimental grower diet (PAG1320, Nutricia, route de Saint Sever, Haut Mauco, BP27, France).

Commercial Diet PAG 1320		
Category	Constituent / Additive	Quantity
Analytical Constituents	Dry Matter (DM)	87.18%
	Crude Protein	16.45% DM
	Crude Fat	2.25% DM
	Crude Ash	4.57% DM
	Starch	48.52% DM
	Energy (kJ.g^−1^)	18.45

At 10 weeks of age, ducks were randomly allocated into fed or fasted groups (*n* = 90 per group). Following an overnight 12 h fasting period, baseline samples (T0) were collected at 08:00 h. Fed ducks were offered ad libitum access to the grower diet for 10 min, after which feeders were removed. Subsequently sampling was conducted at T20 min, T2 h, T6 h, and T12 h for both groups, following the postprandial kinetic scheme described by Lasserre et al. (2026). At each time point, ten ducks per group were euthanized via electronarcosis followed by exsanguination. Blood was processed for plasma isolation (2,000 × g, 10 min, 4 °C) and stored at −20 °C. Mandibles (oral floor mucosa) and brains were collected, snap-frozen in liquid nitrogen, and kept at −80 °C. Hypothalamic microdissections and whole-brain homogenizations were performed for gene expression and neurochemical analyses, respectively.

### Biochemical assays

Circulating chemerin concentrations were quantified in duplicate using a commercial chicken-specific enzyme-linked immunosorbent assay **(ELISA)** (MBS738819, MyBioSource, USA) according to the manufacturer’s instructions. Optical density was read at 450 nm (Tecan, Magellan, Switzerland). The intra-assay coefficient of variation was <15%.

### RNA extraction and quantitative real-time PCR (RT-qPCR)

Total RNA was extracted from frozen oral floor mucosa and hypothalamic tissues using TRIzol reagent (Invitrogen, Thermo Fisher Scientific). RNA concentration and purity were measured using an EPOCH 2 spectrophotometer (BioTek), and structural integrity was checked on 1% agarose gels. Reverse transcription (1 µg RNA) was carried out using SuperScript III Reverse Transcriptase (Invitrogen), Random Primers, and dNTPs (Promega). Real-time PCR assays were executed on a CFX384 system (Bio-Rad) using PerfeCTa SYBR Green FastMix (VWR) as detailed by Lasserre et al., 2026. Target gene primers are listed in Table 2. Relative mRNA expression was calculated using the 2^-ΔΔCt^ method (Livak and Schmittgen, 2001), with ***EF1*** (Elongation factor 1 alpha) and ***GAPDH*** (Glyceraldehyde-3-phosphate dehydrogenase) serving as reference genes for normalization against baseline (T0) levels*.* Raw Ct values are provided as Supplementary data.

**Table 2:** Nucleotide sequences of the PCR primers used to evaluate the mRNA abundance of transcripts by real-time quantitative PCR (5′−3′ sens).

**Gene family**	**Gene**	**Forward (5′−3′)**	**Reverse**
Taste receptor	*T1R1*	AGGTCAATGTCACCGTCGTC	ACATCCCAATCACAGAGCCG
	*T1R3*	TCTTGATCCCACAGGTCAGCTA	CCAACGTTGCGCAAGAAGAG
	*T2R9*	CACCTGCGCTGCTCTAAAAAG	TGCTCAGCATAGGATGGCAG
	*T2R40*	ATCCTAGCCAAGAGGCCCAT	CGAAATTTGGGGTTGCTCCA
	*T2R116*	ATGCCCACATCAAAGCCATG	GCTTCACACAGGGCAGAATC
Acid sensing ion channel	*ASIC4*	AGTCCATCTGCTCTCCCAAC	TTCTTGTTGTACTTGCGGGC
	*ASIC1-2*	TAGCCACAGGGGGAGGTATC	ATAGCCTCGGCGGTACCTTG
	*ASIC1-1*	GCACCATTTCCTTCTCCCGTG	CTCCACGAAGATGTGGCTCAG
Otopetrin receptor	*OTOP3*	AGTCTGGATTAGGGGGTCACTG	GCTATGGTCAGCATCAGCCC
Glutamate metabotropic receptor	*GRM3*	AGGAGTCATAGGCGGTTCCT	AACCGTTGACACGTAGGTCC
	*GRM4*	TCGCAGTCGGCAGAAAAGAT	TCCGGGTGGAAGAGGATGAT
G protein receptor	*GPR119*	AACCCCCTGCTCTACTCCTG	TGAGTTCATGGTTGTGGCAGA
Free Fatty acid receptor	*FFAR4*	CCGAGCCCTCATTGTGTTGA	GCCCCATTCCCTGTATGGTT
	*FFAR3*	GCCCAAGTAGCTCTGGAGTC	GCCATTGGGGACAGAGATGT
Lysophosphatidic acid receptor	*LPAR1*	GGTCATTGTGCTTGGTGCTTT	AGGATCTGCTTGAAGGTCGC
	*LPAR3*	GCGGGGCTGCGGGCACGG	GGAGTGTCCTAAACCTCGTT
	*CD36*	TTGAAGGCCTGAGCCCAAAT	GGCTGCCTGTGCATTGAATC
Stromal interaction molecule	*STIM1*	CTCCCATGTCCATACAATCTCC	AAAAAGCCAGGCTGCATATTGTA
Calcium release-activated calcium modulator	*ORAI2*	GGAAGTGGGTGGTCACGAAA	AGCCCTATTCTTCCTGCCGT
	*ORAI1*	GCTATGGTGGAAGTGCAACTAG	CTTGCTGAATTTGCACGGCT
G protein alpha subunit	*GNAT2*	TACCTGGCCCCCACTTTTTC	TGGGGTCTGTGTGCTCTTTG
Calcium sensing receptor	*CASR*	AGACTCCTTGAACCTGGACG	AAAGACGACTGGATGAGGCA
Orexigenic neuropeptides	*NPY*	ATCACCAGGCAGAGGTATGGA	CACCACATCGAAGGGTCTTCA
	*AGRP*	CTGGAACCGCAGGCATCAT	GTTGAAGAAGCGGCAGTAGC
Anorexigenic neuropeptides	*CART*	ACTCCTTCCTCCTCAAGTGC	GGACAGGGAAGAGAACAGCT
	*POMC*	GAGAACGGGGTTTTGGCGTG	GCCGAATTTGTTCCAGCGAA
Melanocortin 4 receptor	*MC4R*	AATGCAAAGGGCCACTCCTC	GTACATGGGCGAATGGAGGT

### Indolamine and catecholamine measurement using UHPLC-FL

Levels of monoamines (5-hydroxytryptamine (**5-HT**), L-3,4-dihydroxyphenylalanine (**L-DOPA**)) and their metabolites (5-hydroxyindoleacetic acid (**5-HIAA**), homovanillic acid (**HVA**)) were quantified in oral floor mucosa and whole brain tissues (*n* = 10/group/time point) using an ACQUITY H-Class PLUS UHPLC system coupled with fluorescence detection (Waters, Milford, MA, USA). Sample processing, tissue homogenization, deproteinization, chromatographic separation on a Luna® PFP(2) column (Phenomenex), and detection settings strictly followed procedures described by Lasserre et al. (2026), adapted from Heraud et al. (2022). Target values were normalized to total soluble protein content assessed via the bicinchoninic acid assay (Sigma-Aldrich).

### Statistical analysis

Data were analyzed using R software (v3.6.2; RStudio). Normality and variance homogeneity were evaluated using Shapiro-Wilk and Levene tests, respectively. Logarithmic transformations were applied when parametric assumptions were violated. Normally distributed datasets were evaluated by two-way ANOVA (fixed factors: nutritional status, time, and their interaction). For post-hoc analysis, Dunnett-adjusted comparisons were used against T0 baseline for RT-qPCR data, whereas Tukey-adjusted pairwise comparisons were applied for neurochemical parameters. Non-parametric datasets were analyzed using the Scheirer-Ray-Hare test followed by Dunn's post hoc test. Statistical significance was set at *P* < 0.05.

## Results

### Postprandial modulation of plasma chemerin concentrations

Plasma chemerin concentrations were measured to assess peripheral postprandial responses to feeding (Fig. 1). Plasma chemerin concentrations were significantly affected by time (*P* < 0.0005), feeding condition (*P* < 0.0005) and their interaction (*P* < 0.005). In fed ducks, plasma chemerin concentrations increased rapidly after meal ingestion and reached their highest values at T20 min. Thereafter, concentrations decreased but remained higher than those observed in most fasted groups. In contrast, in fasted ducks, plasma chemerin concentrations were reduced from T2 h onwards compared with T0 and remained low throughout the rest of the experimental period. Overall, these results indicate a rapid postprandial increase in circulating chemerin in response to feeding, whereas prolonged fasting is associated with reduced plasma chemerin concentrations.

**Fig. 1:**
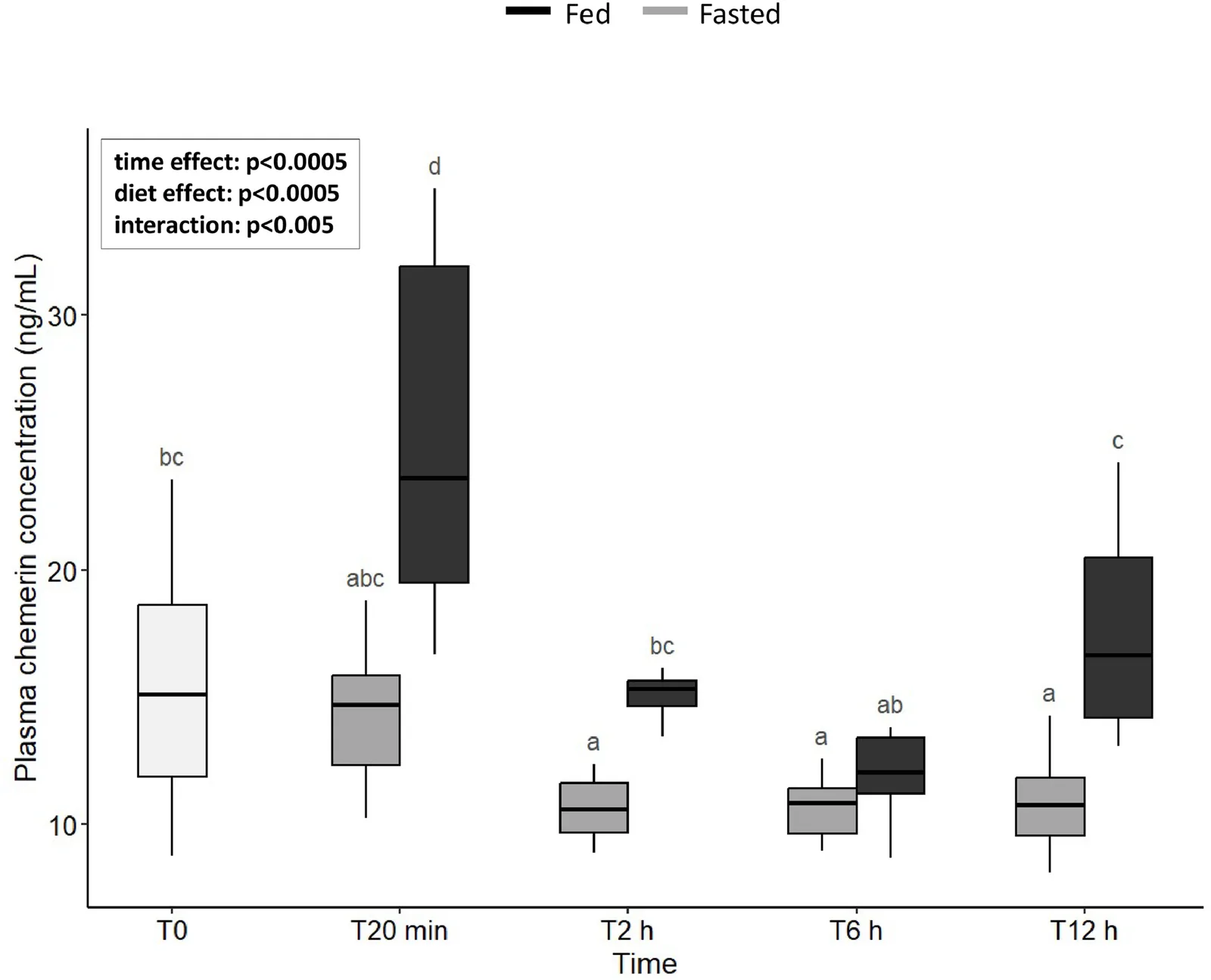
Plasma chemerin concentrations in fed and fasted mule ducks. Data are presented as boxplots (median and IQR). Statistical analyses were performed using two-way ANOVA. Different letters indicate significant differences among groups according to Tukey-adjusted estimated marginal means (*P* < 0.05) (*n* = 10).

### Postprandial regulation of taste receptor genes in the oral floor mucosa

***Sweet and umami receptors.*** Sweet and umami receptor genes displayed limited postprandial modulation overall (Fig. 2, Fig. 3). Expression of ***T1R1*** and ***T1R3*** (Taste receptors type 1 member 1 and 3) was significantly affected by time (*P* < 0.05 and *P* < 0.001, respectively), whereas no effect of feeding condition was detected. *T1R3* expression decreased over time in both the fed and fasted groups. In the fasted group, expression was lower at T2 h (*P* < 0.001) and remained reduced at T12 h (*P* < 0.05), whereas in the fed group a significant decrease was only observed at T12 h (*P* < 0.05). In contrast, *T1R1* expression remained relatively stable, except for an increase at T6 h in the fasted group compared with T0 (*P* < 0.01). Similarly, ***GRM4*** (Metabotropic glutamate receptor 4) expression was affected by time (*P* < 0.05), with decreased expression observed at T12 h in the fed group compared with T0 (*P* = 0.045). No effect of feeding condition or interaction was detected for *GRM4*. ***GRM3*** (Metabotropic glutamate receptor 3) expression remained stable across postprandial time points and feeding conditions.

**Fig. 2:**
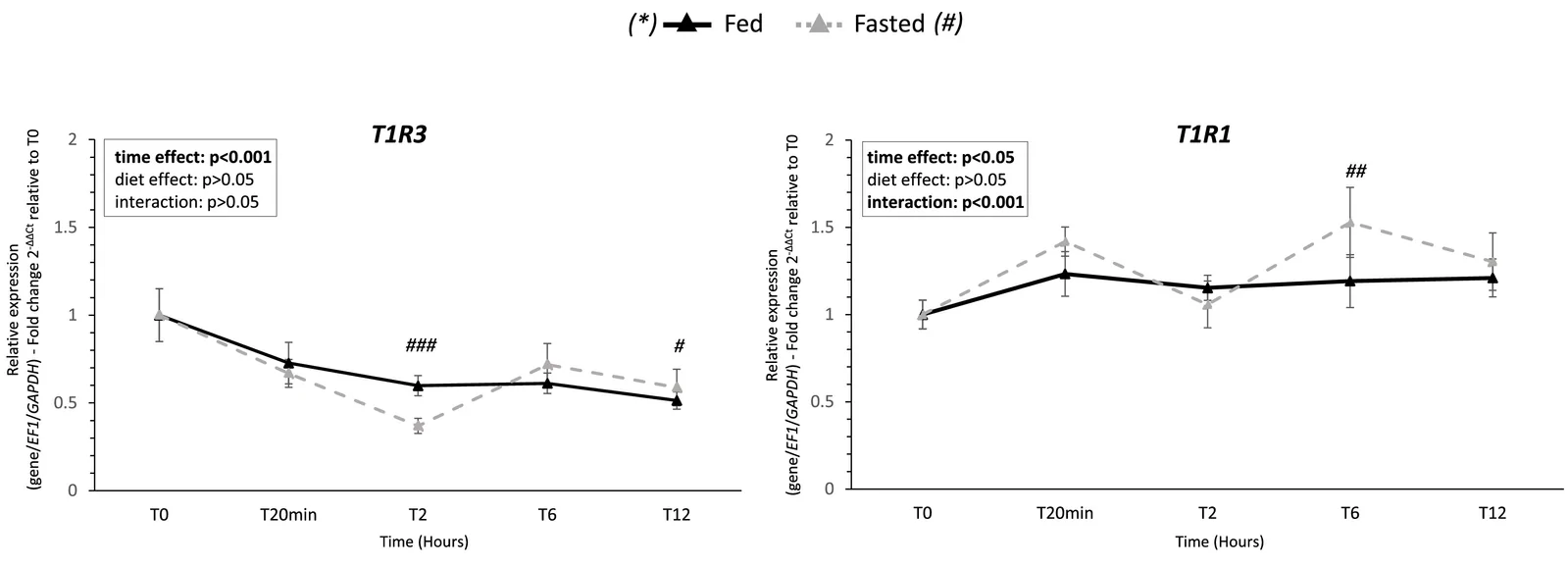
Sweet taste receptor gene expression (*T1R3* and *T1R1*) in the oral floor mucosa of fed and fasted mule ducks. Data are presented as mean ± SEM. Fold changes were calculated using the 2^-ΔΔCt^ method relative to T0. Statistical analyses were performed using two-way ANOVA. When a significant time effect was detected, post hoc comparisons with T0 were performed using Dunnett-adjusted estimated marginal means. Asterisks indicate significant differences compared with T0 within fed ducks, whereas hash symbols indicate significant differences compared with T0 within fasted ducks (*P < 0.05, **P < 0.01, ***P < 0.001; #*P* < 0.05, ##*P* < 0.01, ###*P* < 0.001) (*n* = 10).

**Fig. 3:**
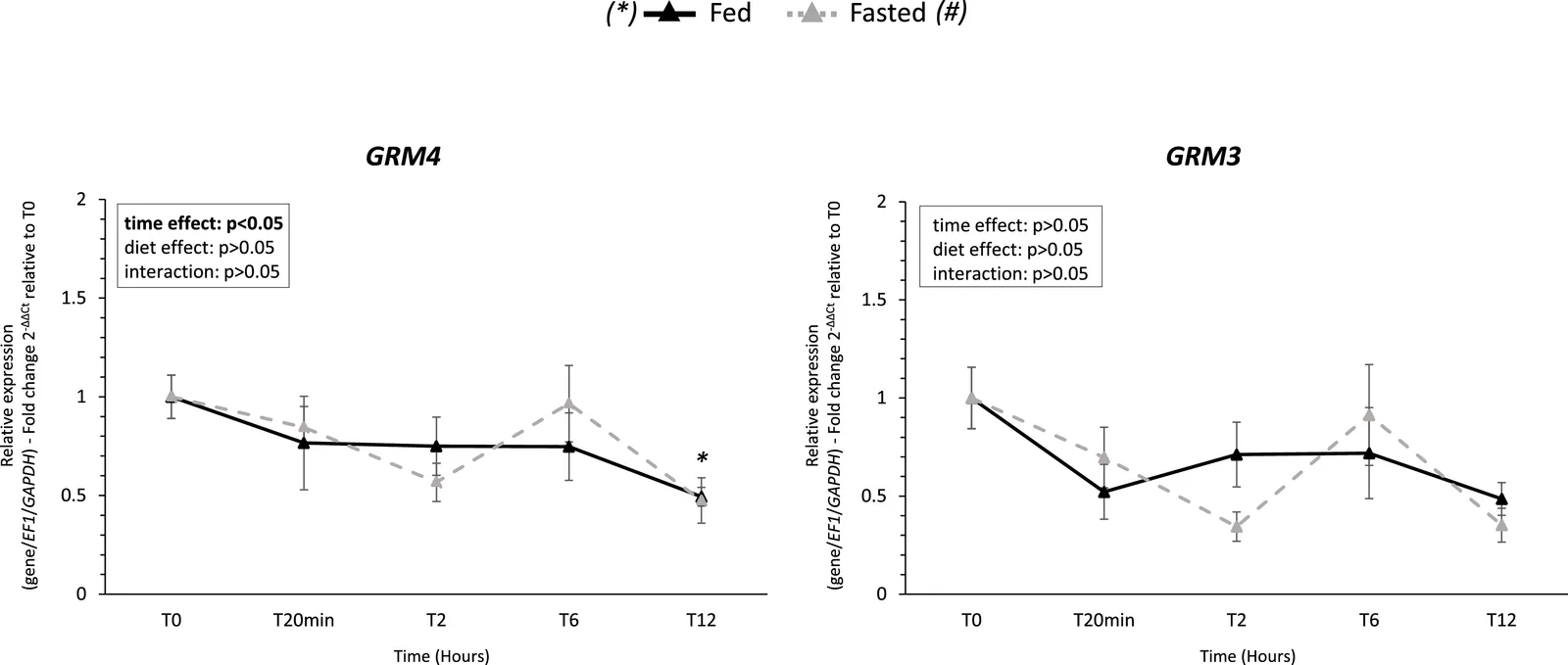
Umami receptor gene expression (*GRM4* and *GRM3*) in the oral floor mucosa of fed and fasted mule ducks. Data are presented as mean ± SEM. Fold changes were calculated using the 2^-ΔΔCt^ method relative to T0. Statistical analyses were performed using two-way ANOVA. When a significant time effect was detected, post hoc comparisons with T0 were performed using Dunnett-adjusted estimated marginal means. Asterisks indicate significant differences compared with T0 within fed ducks, whereas hash symbols indicate significant differences compared with T0 within fasted ducks (*P < 0.05, **P < 0.01, ***P < 0.001; #*P* < 0.05, ##*P* < 0.01, ###*P* < 0.001) (*n* = 10).

***Bitter taste receptors.*** Bitter taste receptor genes showed heterogeneous postprandial responses (Fig. 4). ***T2R9*** and ***T2R40*** (Taste receptors type 2 member 9 and 40) expression remained stable across postprandial time points and feeding conditions, with no significant effects detected. In contrast, ***T2R116*** (Taste receptor type 2 member 116) expression was significantly affected by time (*P* < 0.05) and by the time × feeding interaction (*P* < 0.001). In the fed group, *T2R116* expression decreased at T20 min compared with T0 (*P* < 0.001). In the fasted group, *T2R116* expression decreased at T2h compared with T0 (*P* < 0.001).

**Fig. 4:**
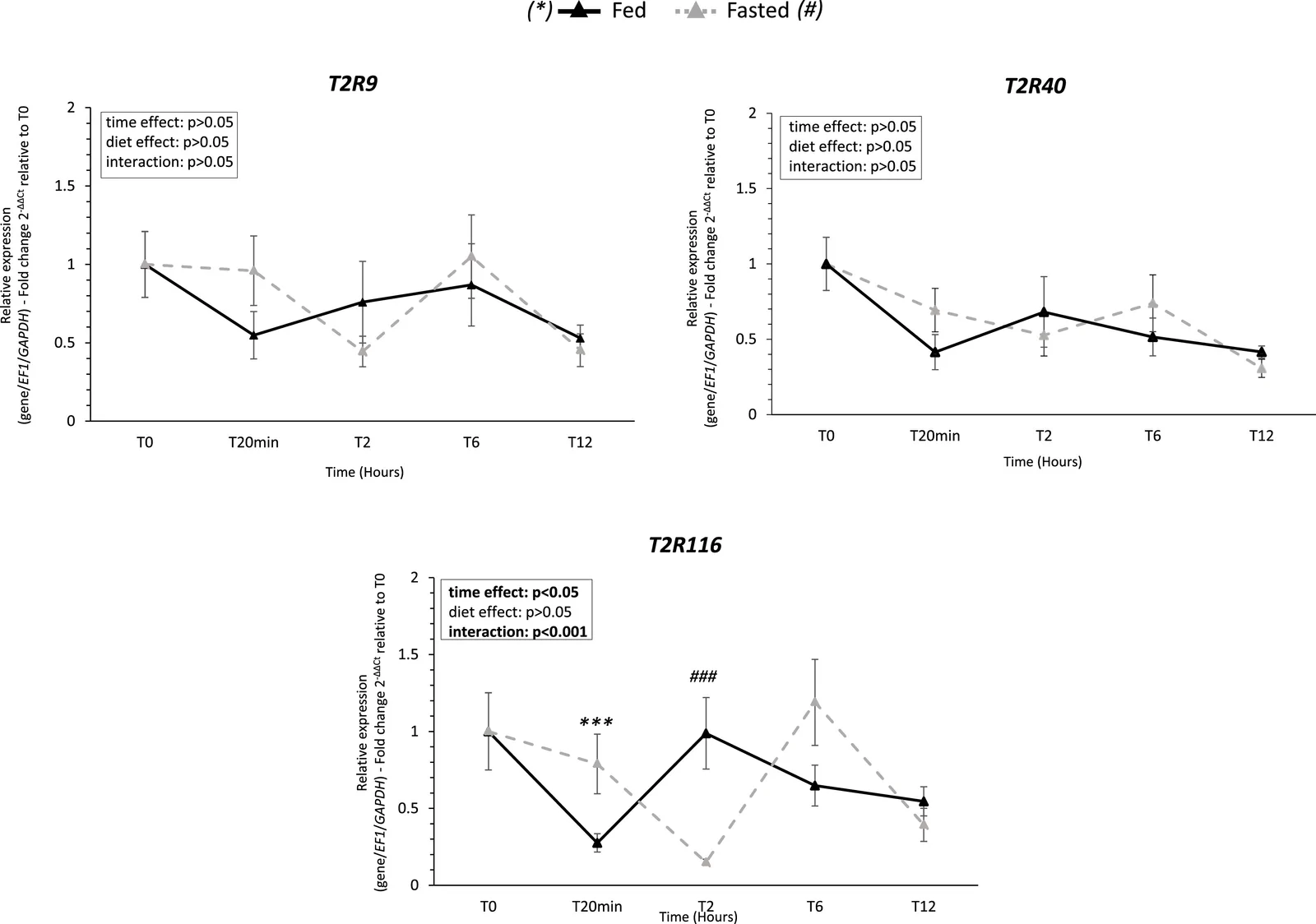
Bitter taste receptor gene expression (*T2R9, T2R40*, and *T2R116*) in the oral floor mucosa of fed and fasted mule ducks. Data are presented as mean ± SEM. Fold changes were calculated using the 2^-ΔΔCt^ method relative to T0. Statistical analyses were performed using two-way ANOVA. When a significant time effect was detected, post hoc comparisons with T0 were performed using Dunnett-adjusted estimated marginal means. Asterisks indicate significant differences compared with T0 within fed ducks, whereas hash symbols indicate significant differences compared with T0 within fasted ducks (*P < 0.05, **P < 0.01, ***P < 0.001; #*P* < 0.05, ##*P* < 0.01, ###*P* < 0.001) (*n* = 10).

***Acid-sensing receptors.*** Among acid-sensing receptors, *ASIC* genes exhibited marked temporal regulation (Fig. 5). ***ASIC1-1*** (Acid-sensing ion channel 1-1) expression was significantly affected by time (*P* < 0.01), with decreased expression observed in the fed group at T20 min (*P* < 0.01) and T12h (*P* < 0.05) compared with T0, whereas no significant changes were detected in the fasted group. ***ASIC1-2*** (Acid-sensing ion channel 1-2) expression was strongly modulated over time (*P* < 0.001). In the fed group, expression decreased at T20 min (*P* < 0.001), T6h (*P* < 0.01), and T12h (*P* < 0.01) compared with T0, while in the fasted group decreases were observed at T20min (*P* < 0.05), T2h (*P* < 0.05), and T12h (*P* < 0.01). Similarly, ***ASIC4*** (Acid-sensing ion channel 4) expression was significantly affected by time (*P* < 0.001). Post-hoc analysis revealed lower expression levels at T20 min and T12 h compared with T0 in both fed and fasted ducks (*P* < 0.05). In contrast, ***OTOP3*** (Otopetrin 3) expression remained stable across postprandial time points and feeding conditions.

**Fig. 5:**
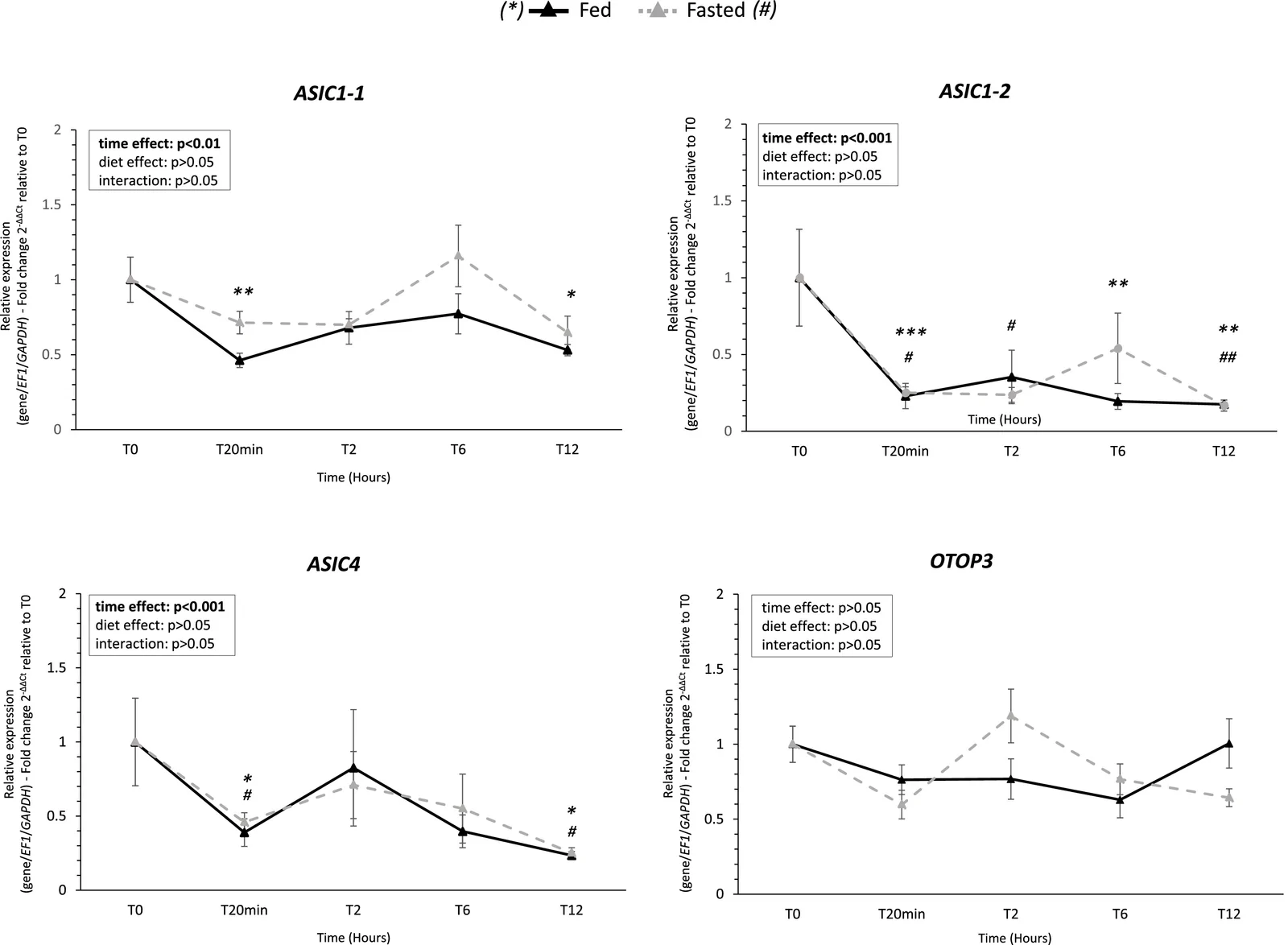
Sour-sensing ion channel gene expression (*ASIC1-1, ASIC1-2, ASIC4* and *OTOP3*) in the oral floor mucosa of fed and fasted mule ducks. Data are presented as mean ± SEM. Fold changes were calculated using the 2^-ΔΔCt^ method relative to T0. Statistical analyses were performed using two-way ANOVA. When a significant time effect was detected, post hoc comparisons with T0 were performed using Dunnett-adjusted estimated marginal means. Asterisks indicate significant differences compared with T0 within fed ducks, whereas hash symbols indicate significant differences compared with T0 within fasted ducks (*P < 0.05, **P < 0.01, ***P < 0.001; #*P* < 0.05, ##*P* < 0.01, ###*P* < 0.001) (*n* = 10).

***Fatty acid receptors.*** Most fatty acid receptor genes showed limited postprandial modulation (Fig. 6). ***CD36*** (Cluster of differentiation 36) expression displayed a significant time × feeding interaction (*P* < 0.05), although post-hoc analyses did not reveal significant differences relative to T0 within either feeding condition. ***FFAR3*** (Free fatty acid receptor 3) and ***LPAR1*** (Lysophosphatidic acid receptor 1) expression were significantly affected by time (*P* < 0.05), but no significant post-hoc differences relative to T0 were identified. In contrast, ***GPR119*** (G protein-coupled receptor 119), ***FFAR4*** (Free fatty acid receptor 4), and ***LPAR3*** (Lysophosphatidic acid receptor 3) expression remained stable across postprandial time points and feeding conditions.

**Fig. 6:**
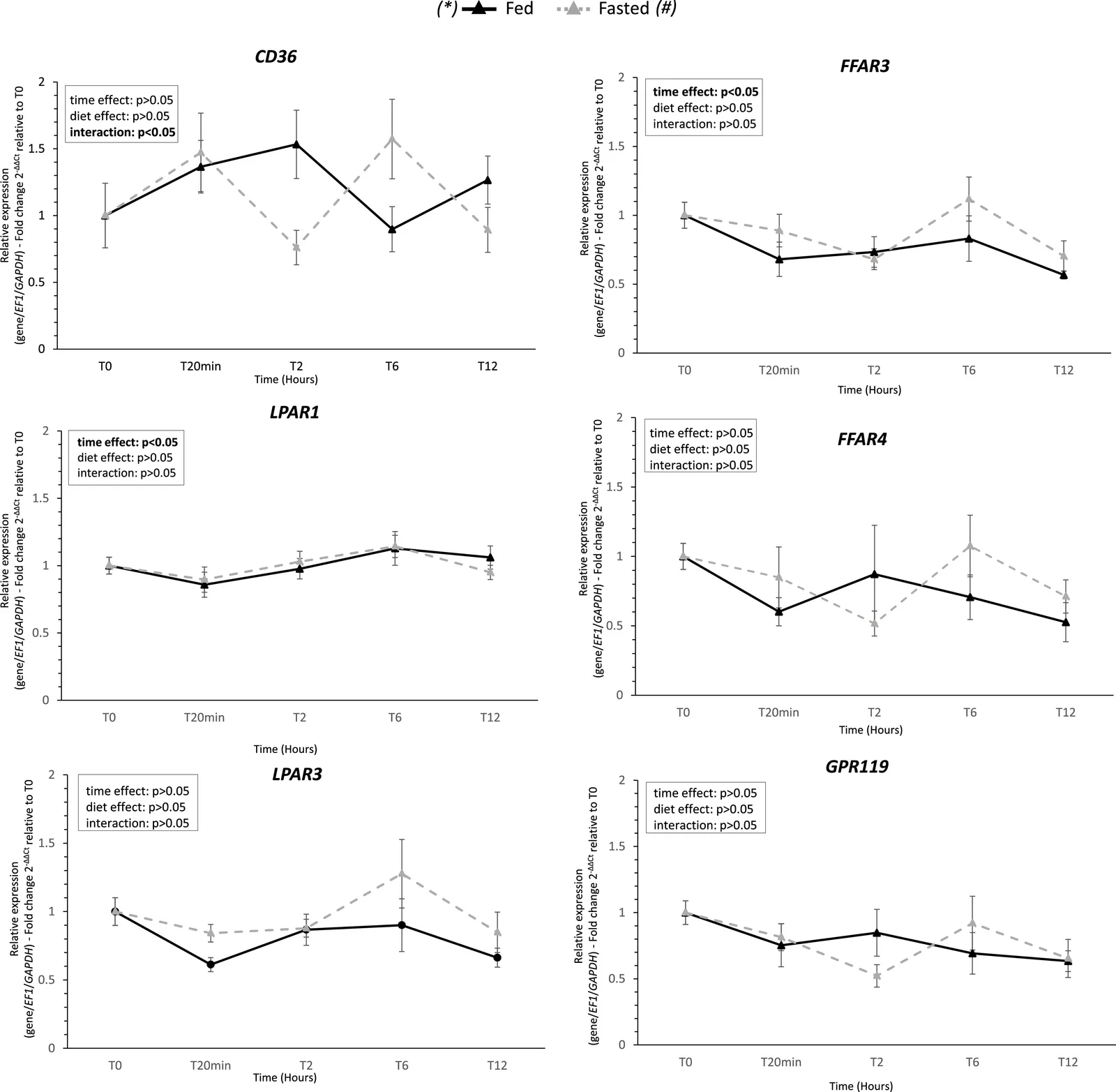
Fatty acid receptor gene expression (*CD36, FFAR3, LPAR1, FFAR4, LPAR3 and GPR119*) in the oral floor mucosa of fed and fasted mule ducks. Data are presented as mean ± SEM. Fold changes were calculated using the 2^-ΔΔCt^ method relative to T0. Statistical analyses were performed using two-way ANOVA. When a significant time effect was detected, post hoc comparisons with T0 were performed using Dunnett-adjusted estimated marginal means.

### Postprandial regulation of calcium-signaling genes in the oral floor mucosa

Among calcium-signaling genes, ***CASR*** (Calcium-sensing receptor) expression was significantly affected by time (*P* < 0.05, Fig. 7). Post-hoc analyses revealed a transient increased in *CASR* expression at T6 h compared with T0 in the fasted group (*P* < 0.05), whereas no significant variations over time were observed in fed ducks. In contrast, ***STIM1*** (Stromal interaction molecule 1), ***GNAT2*** (G protein subunit alpha transducin 2), ***ORAI1***, and ***ORAI2*** (ORAI calcium release-activated calcium modulator 1 and 2) expression remained stable across postprandial time points and feeding conditions.

**Fig. 7:**
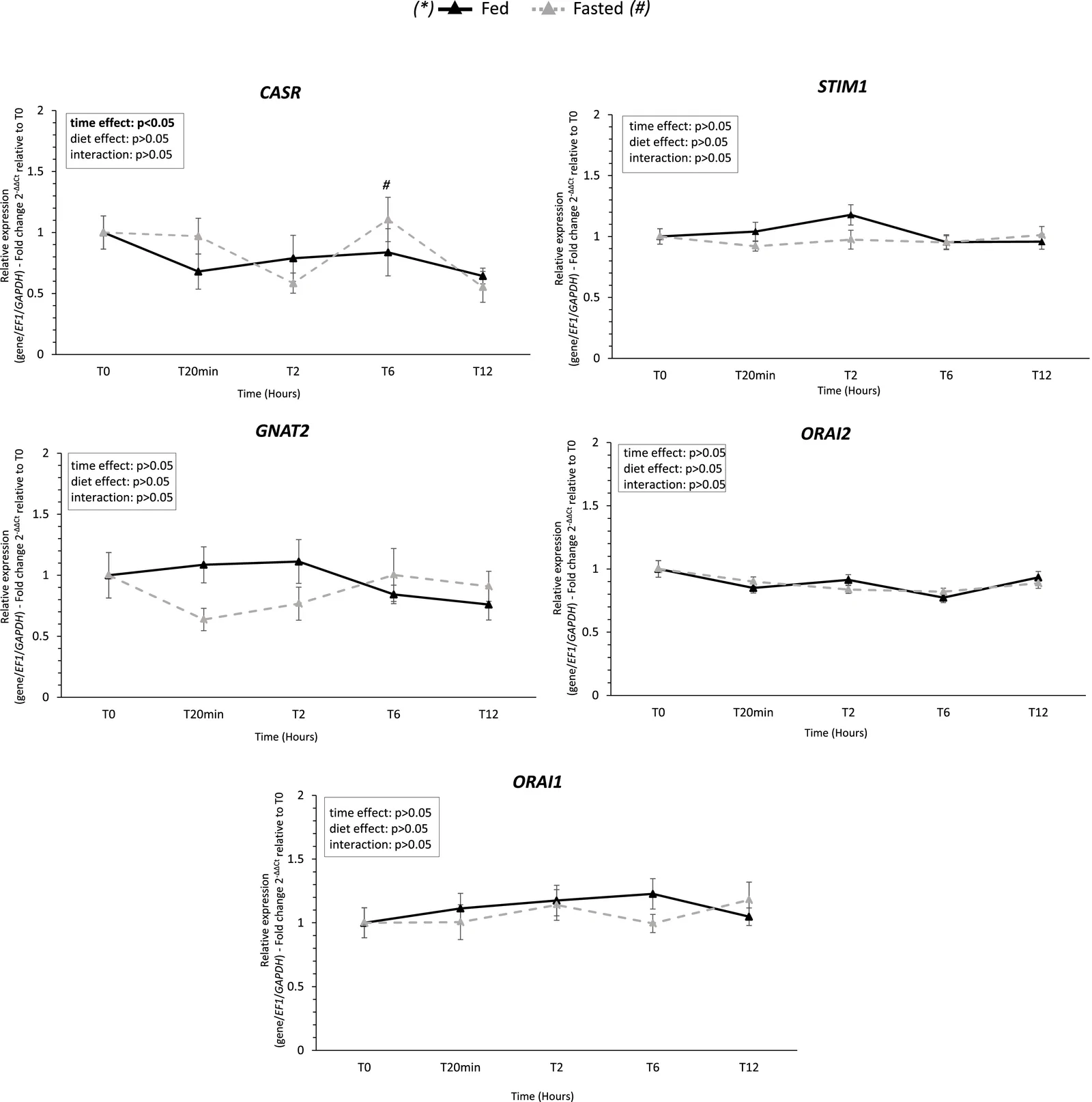
Calcium-signaling and taste-transduction gene expression (*CASR, STIM1, GNAT2, ORAI2*, and *ORAI1*) in the oral floor mucosa of fed and fasted mule ducks. Data are presented as mean ± SEM. Fold changes were calculated using the 2^-ΔΔCt^ method relative to T0. Statistical analyses were performed using two-way ANOVA. When a significant time effect was detected, post hoc comparisons with T0 were performed using Dunnett-adjusted estimated marginal means. Asterisks indicate significant differences compared with T0 within fed ducks, whereas hash symbols indicate significant differences compared with T0 within fasted ducks (*P < 0.05, **P < 0.01, ***P < 0.001; #*P* < 0.05, ##*P* < 0.01, ###*P* < 0.001) (n = 10).

### Postprandial kinetics of serotonin metabolism in oral floor mucosa

Oral serotonin metabolism exhibited marked postprandial modulation (Fig. 8). 5-HT concentrations were significantly affected by time (*P* < 0.005) and by the time × feeding interaction (*P* < 0.005). In fed ducks, 5-HT concentrations increased after meal ingestion, remained elevated at T2 h and T6 h, and returned toward baseline values at T12 h. In contrast, fasted ducks exhibited an early decrease at T20 min followed by increased 5-HT concentrations at T2 h and T6 h. At T20 min, 5-HT concentrations were significantly higher in fed ducks than in fasted ducks.

**Fig. 8:**
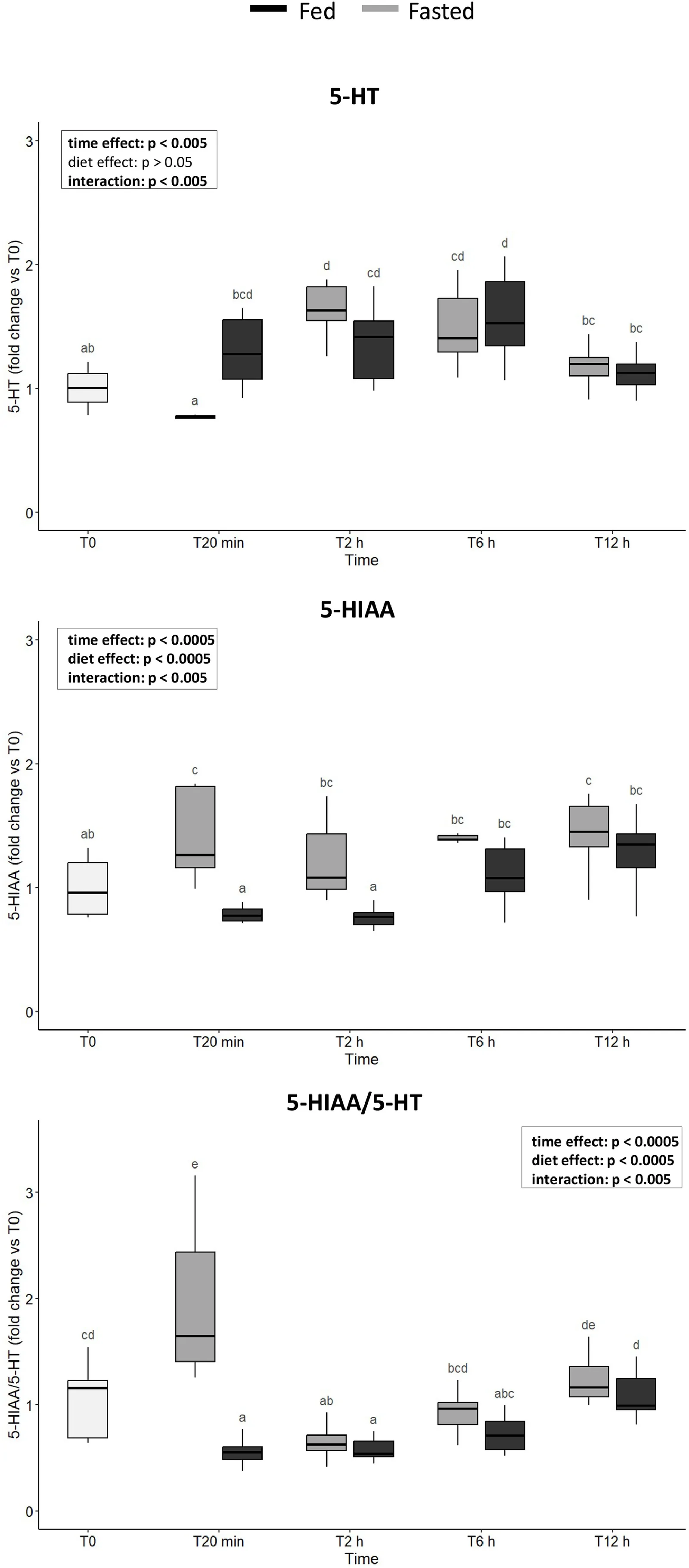
Feeding effects on serotonin turnover in the oral floor mucosa of mule ducks. Indolamine concentrations (5-HT, 5-HIAA and the 5-HIAA/5-HT ratio) in fed and fasted mule ducks at T0, T20min, T2h, T6h and T12h. Data are presented as boxplots (median and IQR). Fold changes are expressed relative to T0. Statistical analyses were performed using two-way ANOVA. Different letters indicate significant differences among groups according to Tukey-adjusted estimated marginal means (*P* < 0.05) (*n* = 10).

Similarly, 5-HIAA concentrations were significantly affected by time (*P* < 0.0005), feeding condition (*P* < 0.0005), and their interaction (*P* < 0.005). Fasted ducks exhibited significantly higher 5-HIAA concentrations than fed ducks at T20 min and T2 h. Thereafter, no significant differences between nutritional conditions were detected.

Consequently, the 5-HIAA/5-HT ratio was significantly affected by time (*P* < 0.0005), feeding condition (*P* < 0.0005), and their interaction (*P* < 0.005). In fed ducks, the ratio markedly decreased immediately after meal ingestion, remained low at T2 h, and progressively returned to baseline values by T12 h. Conversely, fasted ducks exhibited a marked increase in the ratio at T20 min, followed by a decrease at T2 h and a subsequent rise toward T12 h.

### Postprandial kinetics of hypothalamic genes involved in feed intake regulation

*Orexigenic pathway.* Hypothalamic orexigenic markers showed stable or subtle postprandial changes (Fig. 9.**A**). *NPY* expression was significantly affected by time (*P* < 0.05), indicating temporal modulation across the postprandial period, although post-hoc analyses did not isolate specific differences relative to T0. In contrast, *AgRP* expression remained stable across postprandial time points and feeding conditions, with no significant effects of time, feeding status, or time × feeding interaction detected.

**Fig. 9:**
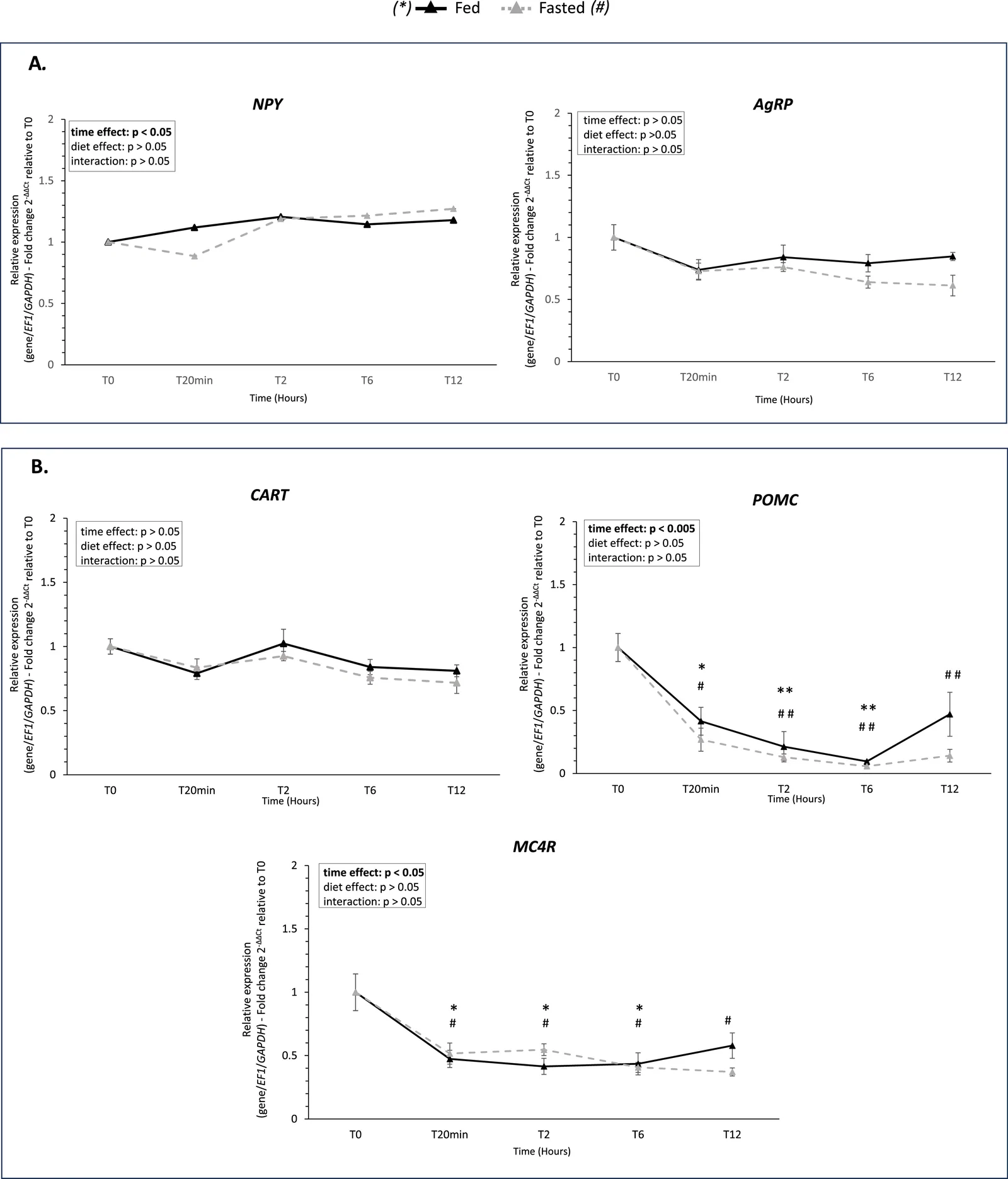
Hypothalamic expression of feed intake regulation genes in fed and fasted mule ducks. (A) Orexigenic genes (*NPY* and *AgRP*). (B) Anorexigenic genes (*CART, POMC*, and *MC4R*). Data are presented as mean ± SEM. Fold changes were calculated using the 2^-ΔΔCt^ method relative to T0. Statistical analyses were performed using two-way ANOVA. When a significant time effect was detected, post hoc comparisons with T0 were performed using Dunnett-adjusted estimated marginal means. Asterisks indicate significant differences compared with T0 within fed ducks, whereas hash symbols indicate significant differences compared with T0 within fasted ducks (*P < 0.05, **P < 0.01, ***P < 0.001; #*P* < 0.05, ##*P* < 0.01, ###*P* < 0.001) (*n* = 10).

*Anorexigenic pathway.* Among hypothalamic anorexigenic markers (Fig. 9.**B**), *CART* expression remained stable across all experimental conditions. In contrast, *POMC* expression was strongly affected by time (*P* < 0.005). Post-hoc analysis revealed that *POMC* mRNA levels significantly decreased from T20 min to T6 h in both fed and fasted (*P* < 0.05 at T20 min, *P* < 0.01 at T2 h and T6 h) ducks compared with T0. At T12 h, *POMC* expression remained significantly depressed only in fasted ducks (*P* < 0.01), while it returned toward baseline levels in fed ducks. Similarly, ***MC4R*** (Melanocortin 4 receptor) expression displayed a significant effect of time (*P* < 0.05). Compared with T0, a significant decrease in expression was observed in both groups at T20 min, T2 h, and T6h (*P* < 0.05). This downregulation remained significant (*P* < 0.05) at T12 h only in fasted ducks.

### Postprandial kinetics of serotonin and L-DOPA metabolism in brain tissue

Brain serotonin metabolism exhibited marked postprandial regulation (Fig. 10**A**). For 5-HT concentrations, significant effects of time (*P* < 0.05), feeding status (*P* < 0.0005), and their interaction (*P* < 0.001) were observed. In fed ducks, 5-HT concentrations increased significantly following meal ingestion, reaching their highest levels at T20 min and remaining elevated at T6 h compared with T0. Moreover, fed ducks exhibited significantly higher 5-HT concentrations than fasted ducks at T20 min, T6 h, and T12 h.

**Fig. 10:**
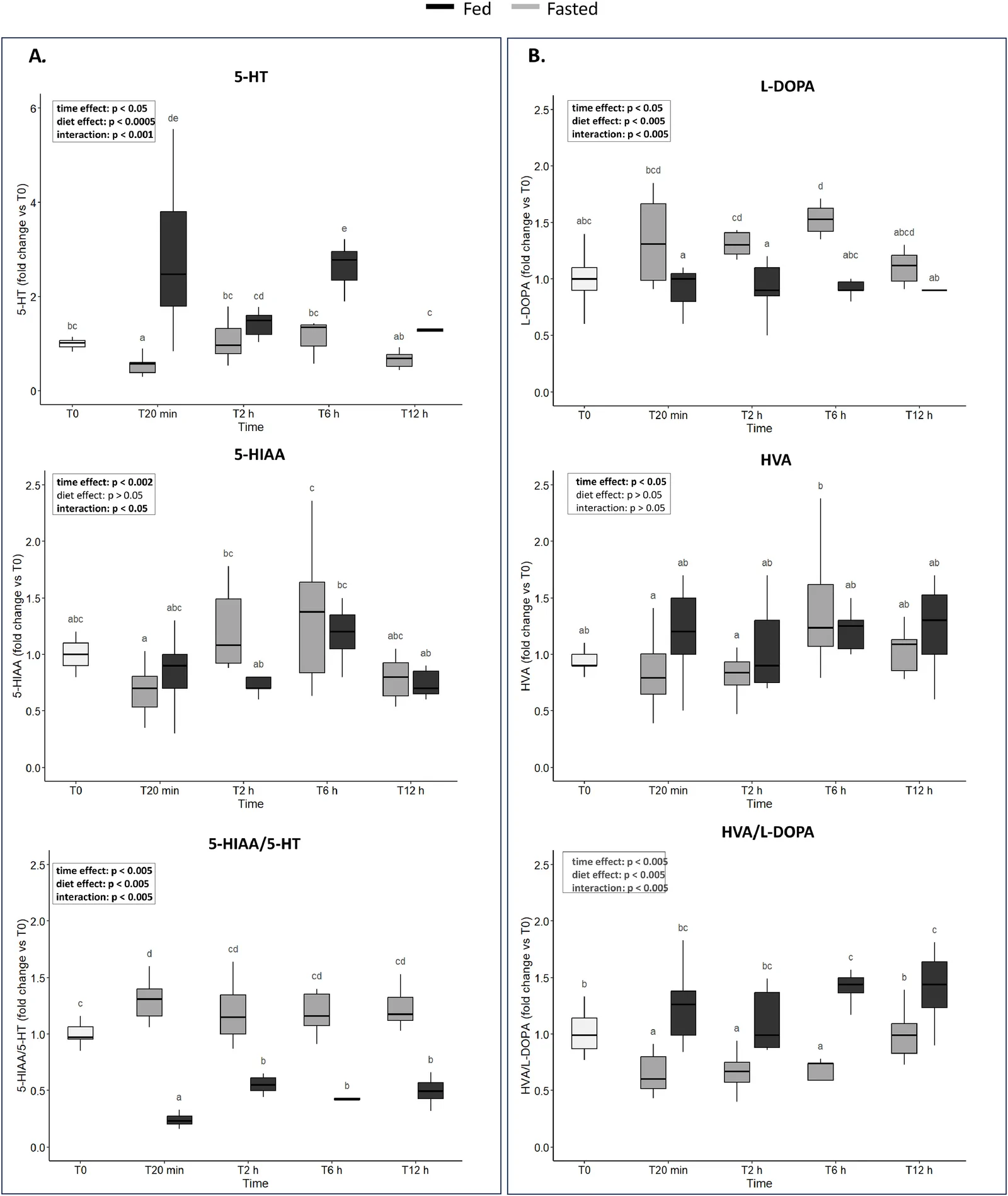
Influence of feeding on brain serotonin and dopamine metabolism in mule ducks. (A) Serotonergic pathway: 5-HT, 5-HIAA, and the 5-HIAA/5-HT ratio. (B) Dopaminergic pathway: L-DOPA, HVA, and the HVA/L-DOPA ratio. Indolamine concentrations (left: 5-HT, 5-HIAA and the 5-HIAA/5-HT ratio) and catecholamine metabolites (right: L-DOPA, HVA and the HVA/L-DOPA ratio) in fed and fasted mule ducks at T0, T20 min, T2 h, T6 h and T12 h. Data are presented as boxplots (median and IQR). Fold changes are expressed relative to T0. Statistical analyses were performed using two-way ANOVA. Different letters indicate significant differences among groups according to Tukey-adjusted estimated marginal means (*P* < 0.05) (*n* = 10).

For 5-HIAA, significant effects of time (*P* < 0.002) and the time × feeding status interaction (*P* < 0.05) were detected, whereas no overall effect of feeding status was observed. The highest 5-HIAA concentrations were observed in fasted ducks at T2 h and T6 h. Accordingly, fasted ducks exhibited significantly higher 5-HIAA concentrations than fed ducks at T20 min and T2 h.

Consequently, the 5-HIAA/5-HT ratio was significantly affected by time (*P* < 0.005), feeding status (*P* < 0.0005), and their interaction (*P* < 0.005). Fasted ducks exhibited significantly higher 5-HIAA/5-HT ratios than fed ducks at all postprandial sampling times (T20 min, T2 h, T6 h, and T12 h).

In the dopaminergic pathway (Fig. 10**B**), L-DOPA concentrations were significantly affected by time (*P* < 0.05), feeding status (*P* < 0.005), and their interaction (*P* < 0.005). The highest L-DOPA concentrations were observed in fasted ducks between T20 min and T6 h, and fasted ducks exhibited significantly higher L-DOPA concentrations than fed ducks at T20 min, T2 h, and T6 h. Conversely, HVA concentrations were significantly affected by time only (*P* < 0.05), with neither feeding status nor the time × feeding status interaction reaching significance. Finally, the HVA/L-DOPA ratio was significantly affected by time (*P* < 0.005), feeding status (*P* < 0.005), and their interaction (*P* < 0.005). Fed ducks exhibited significantly higher HVA/L-DOPA ratios than fasted ducks from T20 min to T12 h.

## Discussion

### Characterization of the oral floor mucosa as a nutrient-sensing hub

The present study provides the first extensive characterization of nutrient-sensing and taste-related receptors in the buccal floor mucosa of ten-week-old mule ducks at the pre-force-feeding stage. Based on our previous tissue screening in four-week-old ducks, the buccal floor mucosa was identified as the region displaying the highest expression levels of chemosensory markers compared with the tongue and palate (Lasserre et al., 2026). Consequently, this tissue was selected as the target region for the present study. A broad repertoire of receptors involved in sweet, umami, bitter, sour and lipid sensing was identified, supporting the hypothesis that the buccal floor mucosa acts as a key site for early chemosensory detection. The identification of multiple gustatory and nutrient-sensing receptors in this tissue therefore extends current knowledge of avian oral chemosensation and highlights the conservation of nutrient-sensing pathways previously described in mammals and other bird species (Roura et al., 2013; Niknafs et al., 2023).

In mammals and birds, T1R receptors are involved in sweet and umami detection, whereas T2R receptors mediate bitter compound sensing, highlighting the evolutionary conservation of gustatory signaling pathways across vertebrates (Zhao et al., 2003; Chandrashekar et al., 2006; Roura et al., 2013; Niknafs et al., 2023). Similarly, ASIC channels and lipid-related receptors, including CD36 and FFARs, have been implicated in sour and fatty acid sensing in birds (Roura et al., 2013; Niknafs et al., 2023). The molecular specialization of the buccal floor mucosa, which displays a distinct chemosensory profile compared with the tongue and palate, as shown during early development in ducks (Lasserre et al., 2026), may also be related to the functional anatomy of the avian oral cavity. In birds, taste buds are predominantly distributed within the palate and mandibular mucosa, enabling direct interaction with ingested feed during oral processing (Gentle, 1972; Kudo et al., 2008). In ducks, feeding mechanics are characterized by limited mastication and rapid swallowing, which may promote efficient exposure of the buccal floor mucosa to dissolved dietary compounds during feed intake and oral processing (Kooloos and Zweers, 1991; West et al., 2022).

### Developmental maturation of oral nutrient-sensing pathways

While most receptor genes displayed limited modulation according to feeding condition, several were significantly affected by time. The frequent temporal effects observed independently of feeding condition further suggest that part of the transcriptional dynamics detected in the oral floor mucosa may reflect general physiological or metabolic regulation rather than meal-specific responses. Compared with previous observations obtained in four-week-old ducks where a marked postprandial transcriptional regulation was widespread in this tissue (Lasserre et al., 2026), postprandial transcriptional modulation of gustatory receptor genes appeared more limited at ten weeks of age. These observations suggest that oral nutrient-sensing pathways undergo developmental changes during growth in ducks. In early stages (four weeks), during growth, the duck's oral mucosa exhibits transcriptional plasticity to optimize nutrient detection and intake. At ten weeks of age, the system appears to have reached a more stable homeostatic state: the receptor repertoire seems largely established, with expression patterns following temporal or circadian dynamics throughout the day rather than showing marked meal-induced transcriptional changes. These findings suggest that oral nutrient-sensing pathways may undergo developmental changes during growth, with gustatory receptor responsiveness appearing more pronounced at earlier stages (Roura et al., 2013; Niknafs et al., 2023).

Importantly, the lack of transcriptional modulation does not necessarily imply a lack of physiological responsiveness. In vertebrate taste systems, sensory regulation may occur through rapid functional mechanisms including post-translational modifications, membrane trafficking, neurotransmitter release, and changes in receptor sensitivity rather than *de novo* mRNA synthesis (Chaudhari and Roper, 2010). Similarly, calcium-signaling genes displayed limited responsiveness to feeding condition, both in the present study and in younger ducks previously investigated (Lasserre et al., 2026). In contrast to the absence of postprandial modulation observed for *NPY, AgRP*, and *CART*, a significant and synchronized transcriptional downregulation was detected for the hypothalamic neuropeptides *POMC* and *MC4R*, which characterized a parallel decrease from T20 min to T6 h in both fed and fasted ducks. This synchronized decrease, independent of the immediate nutritional status, points toward a dominant temporal or circadian fluctuation overriding acute meal-induced transcriptional responses during the first hours. Indeed, evidence in mammalian models has established that hypothalamic *POMC* mRNA expression is not static but undergoes marked diurnal oscillations throughout the 24-h cycle (Steiner et al., 1994). This aligns with observations in avian species, where the transcriptional regulation of the central melanocortin system is known to fluctuate based on temporal or environmental factors, often showing a dissociation from feed intake cues (Boswell and Dunn, 2017). Our results suggest that a similar rhythmic decline occurs during the diurnal phase in ducks, synchronizing both the precursor (*POMC*) and its downstream receptor (*MC4R*). Crucially, this temporal kinetic is broken at the late postprandial stage (T12 h), where a divergence occurs between the two experimental groups. While the expressions of both *POMC* and *MC4R* significantly recover toward baseline levels in fed ducks, they remain fully depressed in fasted animals. This late rebound in fed ducks suggests that once the immediate circadian influence wanes, long-term metabolic signaling takes over, upregulating the anorexigenic melanocortin pathway to sustain satiety. Together, these observations suggest that short-term regulation of feed intake in ducks may depend more on rapid functional and neurochemical adaptations than on immediate transcriptional adjustments. This hypothesis is supported by evidence that avian neuroendocrine control of feeding relies on the rapid integration of peripheral and hypothalamic signals (Schwartz et al., 2000; Richards, 2003; Berthoud, 2004; Morton et al., 2006; Arora and Anubhuti, 2006; Denbow, D. M.; Cogan, T., 2022a).

### Selective postprandial responsiveness of CD36 and T2R116

Among the receptors analyzed, the lipid-related *CD36* and the bitter taste receptor *T2R116* stood out as the only two chemosensory markers showing a significant time × feeding interaction. In avian species, lipid metabolism is a primary homeostatic priority, particularly in ducks (Fouad et al., 2018; Bonnefont et al., 2019), thus suggesting that lipid-sensing pathways may be more responsive to feeding status than other gustatory systems. Interestingly, our results demonstrate that this metabolic plasticity is tightly shared with bitter chemosensation through *T2R116*, indicating a specific postprandial coordination between fat detection and bitter-mediated oral evaluation. In vertebrates, CD36 and T2Rs contribute to the oral detection of fatty acids and bitter compounds, participating in feeding-related signaling, toxin avoidance, and anticipatory digestive responses (Roura et al., 2013; Niknafs et al., 2023). In mammals, CD36 has been identified as a major mediator of oral fatty acid detection and cephalic-phase responses associated with lipid sensing and feeding behavior (Gilbertson, 1998; Laugerette, 2005). The potential role of both *CD36* and *T2R116* in triggering localized or cephalic-phase responses in mature ducks therefore warrants further functional investigation.

### Peripheral signaling: chemerin and serotonin

In parallel with these local findings, plasma chemerin concentrations increased specifically following meal ingestion. This postprandial increase supports the hypothesis that chemerin may participate in feeding-related peripheral metabolic signaling in birds. Chemerin is a multifunctional adipokine primarily produced by adipose tissue and involved in energy homeostasis, adipocyte metabolism, and metabolic regulation (Goralski et al., 2007). Previous studies in chickens have shown that chemerin administration reduces feed intake and hypothalamic pathways involved in energy homeostasis, supporting a role for chemerin in avian metabolic regulation (Estienne et al., 2021). The rapid postprandial increase in plasma chemerin observed in the present study further highlights its potential as a marker of feeding status in ducks. Locally, variations in the 5-HIAA/5-HT ratio within the oral floor mucosa indicate rapid serotonin turnover immediately after ingestion. Peripheral serotonergic signaling contributes to taste bud communication and sensory neurotransmission (Zhang et al., 2017; Denbow and Cogan, 2022b). This rapid local serotonergic response may participate in early communication between sensory receptors and afferent pathways before systemic nutrient absorption occurs.

### Postprandial modulation of central monoaminergic pathways

In contrast to the stability of oral receptors, marked modulation was detected in central monoaminergic pathways. In ten-week-old ducks, the elevated central serotonin concentrations and shifted dopaminergic dynamics in fed animals demonstrate a clear postprandial engagement of satiety and reward circuits (Donovan and Tecott, 2013; Voigt and Fink, 2015; Zhang et al., 2017; Denbow and Cogan, 2022b). Across vertebrate species, central serotonergic transmission serves as prominent anorexigenic signal governing hypothalamic intake regulation. Similarly, dopamine is recognized as a key mediator linking nutrient availability, motivational processes, and feeding behavior (Palmiter, 2007; De Araujo et al., 2012; McCutcheon, 2015; Baik, 2021).

In the present study, the lower 5-HIAA/5-HT ratio observed in fed ducks suggests an active phase of serotonin synthesis and release following feed consumption. This pattern may reflect rapid exocytosis of serotonin and/or postprandial synthesis supported by dietary tryptophan availability. Conversely, the higher ratio measured in fasted ducks is consistent with a state in which serotonin turnover predominates in the absence of feeding-related stimulation, resulting in the progressive conversion of residual serotonin into 5-HIAA by monoamine oxidase (MAO).

The dopaminergic system characterized a persistent feeding status effect without time-dependent fluctuations. Fed ducks exhibited an elevated central HVA/L-DOPA ratio compared to Fasted ones, demonstrating evidence of accelerated dopamine turnover. In these animals, the precursor L-DOPA is rapidly converted into dopamine, released into the synaptic cleft, and subsequently metabolized into HVA. This activation reflects the stimulation of hedonic or motivational reward circuits during meal consumption. In contrast, Fasted ducks exhibited an accumulation of L-DOPA combined with low HVA levels, leading to a collapsed ratio. This neurochemical profile demonstrates a deceleration of dopaminergic turnover, indicating that the reward and motivational circuitry enters a quiescent state during fasting. This central neurochemical drive eventually correlates with the transcriptomic divergence at the latest time point (T12 h) at the hypothalamic level on anorexigenic neuropeptides. While *POMC* expression recovered toward baseline levels in fed ducks to restore long-term satiety, it remained depressed in fasted animals. This demonstrates that while short-term transcriptional variations are driven by time-dependent factors, prolonged energy deprivation ultimately overrides these rhythms, maintaining a strict repression of the anorexigenic pathway to stimulate delayed foraging behavior. This discrepancy between peripheral molecular stability (mRNA levels) and intense neurochemical activity (serotonin and dopamine metabolism) further suggests that short-term postprandial regulation in ten-week-old ducks relies primarily on downstream neural signaling and the activation of pre-existing cascades rather than on the acute transcriptional synthesis of new receptor components.

## Conclusion

In conclusion, the present study shows that the oral nutrient-sensing repertoire is broadly established in ten-week-old mule ducks, whereas the short-term postprandial response relies predominantly on rapid neurochemical adaptations rather than on extensive transcriptional remodeling of gustatory pathways. Together with our previous observations in younger ducks, these findings suggest that the developmental plasticity of oral nutrient-sensing pathways is likely higher during early life, whereas later stages may be characterized by a more stable peripheral sensory machinery associated with dynamic central and neurochemical responses to feeding. This developmental pattern raises the possibility that early-life nutritional or sensory interventions could durably shape oral nutrient-sensing pathways and their downstream regulatory networks. Whether such programming effects could enhance voluntary feed intake later in life, particularly during the pre-force-feeding period, remains an important avenue for future research. This supports the existence of an early oral nutrient-sensing network in ducks, potentially contributing to the rapid integration of feeding-related signals before intestinal absorption occurs, and highlights the oral cavity as an important, yet previously underappreciated, component of avian feeding regulation. From a zootechnical perspective, nutritional strategies targeting feed palatability and oral chemosensory stimulation may therefore be particularly relevant during early developmental windows. At later stages, such as the pre-force-feeding period, effective strategies may need to focus on pathways that retain functional plasticity, including lipid and bitter sensing, or on compounds capable of engaging established neurochemical circuits involved in feed intake regulation.

## Author contributions

**Marie Lasserre**: Drafted the initial manuscript, participated in data interpretation, established analytical workflows, and conducted the experimental investigation. **Yan Fericelli** and **Cécile Heraud**: Developed and adapted experimental protocols, conducted statistical evaluations, and processed trial findings. **Laura-Lou Zwick**: Designed analytical methodologies, evaluated data, and actively contributed to manuscript preparation. **Karel Sasso, Sandra Biasutti**, and **Anne Surget**: Optimized trial methodologies, conducted data evaluation, and assessed analytical outcomes. **Joelle Dupont, Marianne Houssier, Stéphane Panserat**, and **Jérôme Roy**: Contributed to study design, supervised protocol implementation, validated analytical results, and critically evaluated the scientific content. **Karine Gontier**: Conceptualized and supervised the overall project, designed experimental procedures, evaluated data, validated findings, and finalized the manuscript. All authors actively participated in reviewing, critically evaluating, and approving the final version of the manuscript.

## Funding

This study was funded by the CD40 (Departmental Council of the Landes; to KG), the Université de Pau et des Pays de l’Adour (UPPA; to ML), the CIFOG (Comité Interprofessionnel des Palmipèdes à Foie Gras; to KG), and the API PHASE program (INRAE; to KG).

## Ethics approval

All animal procedures were conducted in accordance with French national guidelines for animal research and approved by the institutional Animal Welfare Body (SBEA) of the certified Experimental Station for Waterfowl Breeding (INRAE, Artiguères, France; facility agreement no. B40-037-1).

## Consent for publication

Not applicable.

## Author details

Université de Pau et des Pays de l’Adour, INRAE, NUMEA, Mont-de-Marsan, France.

## Disclosures

The authors declare that they have no conflicts of interest. The funders had no role in study design, data collection and analysis, decision to publish, or preparation of the manuscript.

## Data Availability

The data are available on request from the corresponding author.

## Appendix Supplementary materials

Supplementary data: **Additional File 1: C**alibration parameters of UHPLC-FL analysis.

**Additional File 2:** Raw CT gene.
